# Aging Is Positively Associated with Peri-Sinus Lymphatic Space Volume: Assessment Using 3T Black-Blood MRI

**DOI:** 10.3390/jcm9103353

**Published:** 2020-10-19

**Authors:** Mina Park, Jin Woo Kim, Sung Jun Ahn, Yoon Jin Cha, Sang Hyun Suh

**Affiliations:** 1 Department of Radiology, Gangnam Severance Hospital, Yonsei University College of Medicine, Seoul 06273, Korea; to.minapark@yuhs.ac (M.P.); meowkim@yuhs.ac (J.W.K.); suhsh11@yuhs.ac (S.H.S.); 2 Department of Pathology, Gangnam Severance Hospital, Yonsei University College of Medicine, Seoul 06273, Korea; yooncha@yuhs.ac

**Keywords:** glymphatic system, magnetic resonance imaging, meninges, dural lymphatics

## Abstract

Objectives: Aging is a major risk factor for many neurological disorders and is associated with dural lymphatic dysfunction. We sought to evaluate the association of aging with the volume of the peri-sinus lymphatic space using contrast-enhanced 3T T1-weighted black-blood magnetic resonance imaging (MRI). Methods: In this retrospective study, 165 presumed neurologically normal subjects underwent brain MRIs for cancer staging between April and November 2018. The parasagittal peri-sinus lymphatic space was evaluated using contrast-enhanced 3D T1-weighted black-blood MRIs, and volumes were measured with semiautomatic method. We compared the volumes of normalized peri-sinus lymphatic spaces between the elderly (≥65 years, *n* = 72) and non-elderly (*n* = 93) groups and performed multivariate logistic regression analyses to assess if aging is independently associated with the volume of normalized peri-sinus lymphatic spaces. Results: The normalized peri-sinus lymphatic space volume was significantly higher in the elderly than in the non-elderly (mean, 3323 ± 758.7 mL vs. 2968.7 ± 764.3 mL, *p* = 0.047). After adjusting the intracranial volume, age age was the strongest factor independently associated with peri-sinus lymphatic space volume (β coefficient, 28.4 (5.7–51.2), *p* = 0.015) followed by male sex (β coefficient, 672.4 (113.5–1230.8), *p* = 0.019). Conclusions: We found that the peri-sinus dural lymphatic space volume was higher in the elderly group than in the non-elderly group, and the increased peri-sinus lymphatic space was independently associated with aging. These findings indicate that the peri-sinus lymphatic space may be related with the aging process and lymphatic system dysfunction as well.

## 1. Introduction

For decades, the central nervous system has long been considered an immune-privileged organ because of its limited interactions with the immune system [1]. Although the parenchyma of the central nervous system is devoid of lymphatic vasculature, cerebrospinal fluid (CSF) serves as a carrier of solutes and clear brain waste [2]. One of the pathways of CSF clearance is the lymphatic channels along the exiting nerves at the base of the skull [3]. Lymphatic CSF drainage and the interplay between the lymphatic and central nervous systems have recently gained significant attention because true lymphatic vessels have been shown to exist within the dura [4,5]. Subsequently, the impairment of the dural lymphatic pathways has been shown to slow the paravascular efflux of amyloid-β from interstitial brain fluid, and it may represent a factor aggravating Alzheimer’s disease and age-associated cognitive decline [6]. Furthermore, previous animal studies have demonstrated that advanced aging is associated with impaired glymphatic/dural lymphatic clearance [6,7]. Thus, the function of the glymphatic system is presumed to decline with age in relation to dural lymphatic dysfunction.

Recently, Absinta et al. reported that dural lymphatic vessels could be visualized by magnetic resonance imaging (MRI) in both human and non-human primates using high-resolution T2-fluid-attenuated inversion recovery (FLAIR) and T1-weighted black-blood MRI with intravenous gadolinium-based contrast administration [8]. They observed the existence and topography of the lymphatics using immunohistochemical assessments of dural samples and described a possible new method with which to assess the dural lymphatic system. Subsequent human studies, however, demonstrated that the enhancing structure within the parasagittal dura previously presumed to be dural lymphatic vessels may not be true dural lymphatic vessels but a bridging space enabling the CSF-mediated exchange of molecules between dural lymphatic vessels and brain tissue [9,10].

Therefore, we defined the MRI-visible enhancing peri-sinus structure as the peri-sinus lymphatic space, and hypothesized that the volume of the peri-sinus lymphatic space, a subnetwork of the glymphatic system, would be associated with aging in humans. Herein, we measured the volumes of the peri-sinus lymphatic space using gadolinium enhanced T1-weighted black-blood MRI and evaluated the association between the peri-sinus lymphatic space and aging.

## 2. Materials and Methods

### 2.1. Participants

**Figure 1:**
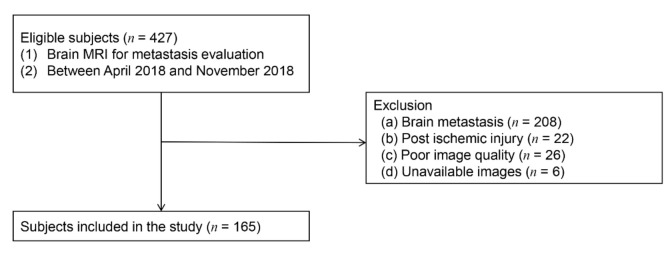
Participant selection flowchart.

The Institutional Review Board of Gangnam Severance Hospital approved this retrospective cross-sectional study and waived any requirement for informed consent because of its retrospective nature. The study included 427 consecutive patients who had suspected or confirmed cancer and who underwent brain MRIs to detect brain metastases at our institution between April and November of 2018. Exclusion criteria for this study included the presence of any known neurologic disorders that might impair blood–brain barrier function (including brain metastases (*n* = 208) or post-ischemic brain injury (*n* = 22)), poor image quality (*n* = 26), and unavailable images (spoiled gradient-recalled (SPGR), T2, and FLAIR images (*n* = 6)). Of the 427 patients, 262 were excluded (Figure 1), yielding a final cohort of 165 presumed neurologically normal patients. We retrospectively retrieved demographic information, including age and sex, as well as histories of underlying cancer, comorbid conditions, hypertension, diabetes mellitus, and chemotherapy statuse from electric medical records. For the current analysis, patients older than 65 years were classified as elderly.

### 2.2. Image Acquisition

All MRI images were obtained using a 3-T MRI scanner (Discovery MR750; GE Healthcare, Milwakee, WI, USA) with a 16-channel head coil and imaging sequences including axial T2-weighted images (T2WI), FLAIR, and 3D SPGR. The parameters for T2WI were as follows: repetition time (TR) = 6380 ms, echo time (TE) = 108 ms, minimal flip angle (FA) = 160 degrees, field of view (FOV) = 250 mm, voxel size = 0.7 × 0.7 × 5.0 mm, turbo factor = 17, and acquisition time = 2:41 min. The axial FLAIR had the following parameters: TR = 8500 ms, TE = 121 ms, inversion time = 2500 ms, FA = 180 degrees, FOV = 230 mm, voxel size = 0.7 × 0.7 × 5.0 mm, acquisition time = 2:52 min, and strong fat saturation. The parameters for the axial 3D SPGR sequence were as follows: TR = 8.3 ms, TE = 3.3 ms, inversion time = 450 ms, FA = 20 degrees, FOV = 220 mm, voxel size = 0.43 × 0.43 × 1 mm, acquisition time = 2:52 min, and strong fat saturation.

In addition, 3D T1-weighted black-blood MRIs were acquired after 3 min and 40 s of administration of a compact bolus (0.2 mmoL/kg) of gadobutrol at an injection rate of 5 mL/s. To obtain 3D T1-weighted black-blood images, a motion-sensitized driven-equilibrium prepared pulse was emitted before the 3D T1-weighted fast spin-echo sequence with a variable flip angle. The scan parameters were as follows: TR = 500 ms, TE = 24.5 ms, slice thickness = 1 mm, echo train length = 24, FA = variable, FOV = 220 mm, voxel size = 0.86 × 0.86 × 1 mm, and acquisition time = 4:50 min.

### 2.3. Image Analyses

A neuroradiologist (M.P.) with 7 years of experience assessed the MRIs for Fazekas and enlarged perivascular space (ePVS) scores. T1- and T2-weighted and FLAIR images were used for visual assessments. White matter hyperintensities (WMHs) were defined as hyperintense white matter lesions on FLAIR images per the Standards for Reporting Vascular Changes in Neuroimaging criteria, and graded in accordance with the Fazekas scale as deep (0 = absent; 1 = punctate; 2 = early confluent; 3 = confluent) and periventricular (0 = absent; 1 = caps or pencil-thin lining; 2 = smooth halo; 3 = irregular WMH extending into the deep white matter) WMHs [11,12]. The quantities of ePVSs in the basal ganglia (BG) and centrum semiovale (CSO) were rated as follows: 0 = no ePVS, 1 = 1–9 ePVS, 2 = 10–20 ePVS, 3 = 21–40 ePVS, and 4 > 40 ePVS [13]. In each case, the most affected hemisphere was rated. ePVSs were defined as small, sharply delineated CSF intensity structures that follow the orientation of the perforating vessels and run perpendicularly to the brain surface [14]. For the analysis, a score of ≥3 was designated as ePVS-positive, and patients were classified into either ePVS (+) or ePVS (-) groups in the BG and CSO.

### 2.4. Segmentation of Dura-Associated Lymphatics

**Figure 2:**
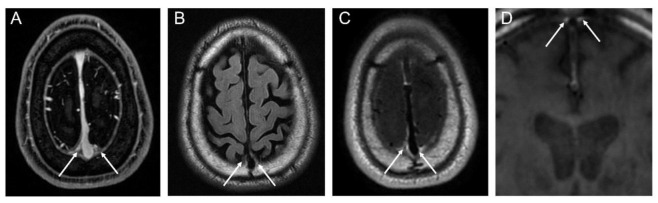
MRI visualization of peri-sinus lymphatic space on conventional contrast-enhanced axial T1-weighted image (**A**), axial FLAIR image (**B**), contrast-enhanced axial T1-weighted black-blood image (**C**), and coronal T1-weighted black-blood image (**D**).

**Figure 3:**
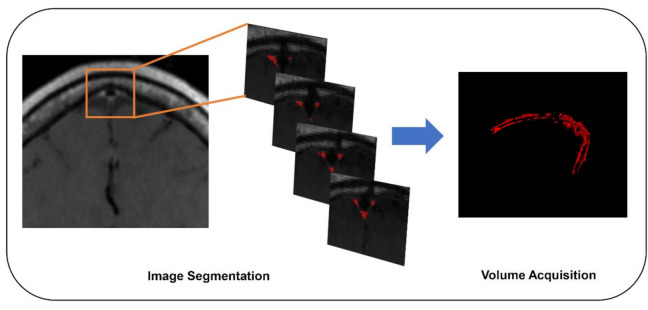
Segmentation workflow of peri-sinus lymphatic space using coronal T1-weighted black-blood MRI.

We identified the peri-sinus lymphatic space (Figure 2) as the enhancing structure located next to the superior sagittal sinus visible on coronal contrast-enhanced 3D T1-weighted black-blood images with high signal intensity on FLAIRs, as suggested by the previous literature [8,15]. The volume of dura-associated lymphatics (Figure 3) was measured from the mid-portion at the eyeball level through to the torcular herophili level by a region of interest (ROI) drawn section-by-section on coronal contrast-enhanced 3D T1-weighted black-blood images using a semiautomatic method with an interactive level-set volume of interest. Threshold-based algorithms were employed using ITK-SNAP and 3D slicer (version 4.9.0; available at: http://slicer.org/software). The ROI was drawn by a neuroradiologist (M.P.) with 7 years of experience who was blinded to all corresponding clinical information. Another neuroradiologist (S.J.A.) with 9 years of experience) drew ROIs in 15 randomly selected patients for the assessment of interrater reliability. The peri-sinus lymphatic volume was normalized with ICV using the residual method, which is known to be less susceptible to systematic and random errors [16].

### 2.5. Brain Volume Analysis

To assess brain volumes, “volBrain” with 3D SPGR was used. volBrain provides automatically generated volumetric information on brain MRIs using an open access platform based on an advanced pipeline that provides the automatic segmentation of several brain structures [17]. Volumes/segmentation and structural asymmetries were also assessed. The total ICV for each patient was used for subsequent analyses.

### 2.6. Statistical Analyses

Continuous parameters are presented as means± standard deviations. Normality was analyzed using the Kolmogorov–Smirnov test to prove normal distribution for all parameters. Independent Student’s *t*-tests were used to compare the mean values of continuous parameters, including peri-sinus lymphatic volume, accordance to aging status. To explore the relationships between peri-sinus lymphatic volume and other clinical continuous data (age, and ICV), Pearson correlation tests were used. In addition, multiple linear regression analyses using the enter method were used to further examine independent factors associated with peri-sinus lymphatic volume. Intraclass correlation coefficients (ICC) were calculated to assess the agreement and repeatability of the measurements of peri-sinus lymphatics in the 15 randomly selected cases. Observer agreement was classified as poor (ICC, 0–0.20), fair (ICC, >0.20–0.40), good (ICC, >0.40–0.75), or excellent (ICC, >0.75) [18]. Data were analyzed using SPSS software (version 25; IBM, Armonk, NY, USA). *p*-values of < 0.05 indicated statistical significance.

## 3. Results

### 3.1. Participant Characteristics

A total of 165 patients (mean age, 62.0 ± 10.9 years; M:F, 91:74) met the study’s inclusion criteria. There were 72 elderly patients (mean age, 71.8 ± 5.2 years; M:F, 46:26) and 93 non-elderly patients (mean age, 54.6 ± 7.7 years; M:F, 45:48).

The mean normalized peri-sinus lymphatic volume for all subjects was 4028.0 ± 1529.8 mm^3^. Normalized peri-sinus lymphatic volumes were significantly higher in the elderly than in the non-elderly group (4417.6 ± 1470.6 mm^3^ vs. 3726.5 ± 1513.9 mm^3^, *p* = 0.004; Table 1). Age was significantly higher in the elderly group (71.8 ± 5.2 years) than in the non-elderly group (54.6 ± 7.7 years, *p* < 0.001). The primary cancer type differed according to the age status (*p* = 0.028). The incidence of hypertension (*n* = 33 (45.8%) vs. *n* = 15 (16.1%), *p* < 0.001) was also higher in the elderly group. Furthermore, total Fazekas scores were also higher in the elderly group than in the non-elderly group (2.9 ± 1.6 vs. 1.5 ± 0.9, *p* < 0.001). High burden ePVS in both the BG (*n* = 15 (20.5) vs. *n* = 3 (3.2%), *p* < 0.001) and the CSO (*n* = 39 (54.9%) vs. 21 (22.6%), *p* < 0.001) were more frequently found in the elderly group than in the non-elderly group. Normalized peri-sinus lymphatic volumes were found to be significantly correlated with age (r = 0.242, *p* = 0.002) and ICV (r = 0.337, *p* < 0.001).

### 3.2. Aging and Its Association with Normalized Peri-Sinus Lymphatic Volume

**Figure 4:**
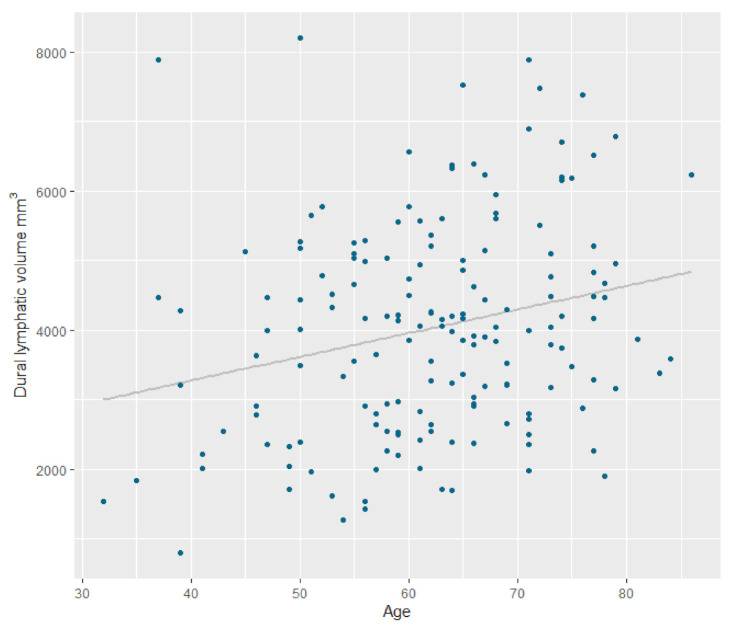
Scatter plot displaying the relationship between dural lymphatic vessel volume and age.

Table 2 summarizes the results of the univariate and multivariate linear regression analyses regarding the associations between variables and normalized peri-sinus lymphatic space volume. After adjustment, multivariate regression analysis showed that age (β coefficient, 28.4 (5.7–51.2), *p* = 0.015 was significantly associated with normalized peri-sinus lymphatic volume (Figure 4). Moreover, male sex (β coefficient, 672.4 (109.9–1234.9), *p* = 0.019) and ICV (β coefficient, 2.3 (0.16–4.4), *p* = 0.035) were also independently associated with normalized peri-sinus lymphatic volume.

### 3.3. Differences Regarding Effects of Aging on Normalized Peri-Sinus Lymphatic Volume Based on Sex

In females, there was no significant linear correlation between age and normalized peri-sinus lymphatic volume after the adjustment of ICV (β coefficient, 2.1 (−30.9 to 35.0), *p* = 0.900), while in males, there was a significant correlation between age and normalized peri-sinus lymphatic volume after the adjustment of ICV (β coefficient, 50.5 (26.4–74.5), *p* < 0.001).

### 3.4. Interrater Reliability of Peri-Sinus Lymphatic Space Volume Measurements

The interrater reliability of peri-sinus lymphatic volume measurements was highly reproducible (ICC, 0.974; 5% confidence interval: 0.922–0.991) between two raters.

## 4. Discussion

In the present study, we found the peri-sinus lymphatic space to be significantly associated with aging even after adjusting for sex and ICV. Recent works have revealed that meningeal lymphatic vessels in the dura serve macromolecular clearing functions in the brain [4,5]. These dural lymphatic vessels are distributed along large blood vessels, such as the superior sagittal sinus, in patterns similar to those observed in the peripheral lymphatic vasculature [8]. A recent study suggested that MRIs using contrast-enhanced FLAIR or black-blood T1 weighted images could be used to assess dural lymphatics, thus highlighting the possibility of the in-depth investigation of both the anatomy and function of in vivo dural lymphatics [8]. Other studies have confirmed that the dural lymphatics in humans can be visualized using 3T contrast-enhanced MRI and that there is, indeed, flow inside the dural lymphatics, supporting the notion that this structure is one with dynamic flow [9,15]. T1-weighted black-blood MRIs selectively suppress the signal of fast moving blood flow and provide a high contrast-to-noise ratio for the contrast material. They consequently offer a higher detection rate of metastasis or slow collateral flow in acute stroke [19,20]. Our results also exhibited strong contrast in the peri-sinus-enhanced space on T1-weighted black-blood MRIs between the superior sagittal sinus and the periosteal layer of the dura, which is in line with previous findings and suggests that slow flow or a low contrast concentration is present in the dural lymphatics. However, according to the recent literature, the enhancing structure within the parasagittal dura may not resemble the previously reported tubular-shaped structures found in true lymphatic vessels with a submillimeter diameter [4,5]. Considering that lymphatic vessels within the dura lack a tight junction and the recent observations of the CSF and molecule drainage in this structure, [9,10] this peri-sinus enhancing space that we measured may represent the space as a bridging link for the CSF-mediated exchange of molecules between the brain tissue and dural lymphatic vessels and not the lymphatic vessels themselves.

Theoretically, dural lymphatics exist along the dural sinus as well as the basal cistern, [21] but dural lymphatic vessels are mainly distributed along large blood vessels, such as the superior sagittal sinus. Therefore, they are best visualized at the peri-superior sagittal sinus [4,8]. Those at other sinuses in the basal cistern are barely distinguishable from adjacent venous and skull structures. However, the dural lymphatic vessels at the base of the skull also have important drainage functions [21]. The whole-brain segmentation of the peri-sinus lymphatic space is an important goal for future research.

Aging is known to negatively affect the function of dural lymphatic vessels. Many mice model studies have shown changes of morphology in the dural lymphatic system as well as the decreased function of dural lymphatic vessels in old mice. Our observations of the increased peri-sinus lymphatic space volume on MRIs in the elderly group have shown that increased peri-sinus lymphatic space volume may be a marker of impaired dural lymphatic drainage. Lymphatic hyperplasia is regarded as a compensatory mechanism that allows for functional adjustments to the capillary lymphatic hypertension in lymphoedema [22]; thus, increased peri-sinus dural lymphatic spaces can be a result of stasis or hyperplasia in the lymphatic system and be associated with impaired lymphatic drainage and thus impaired CSF drainage. These issues may lead to the abnormal accumulation of proteins such as amyloid-β.

Interestingly, males showed a higher peri-sinus lymphatic space volume that females. Moreover, according to the subgroup analysis by sex, the correlation between aging and peri-sinus lymphatic space volume was clearer in males than in females. Sex differences in the dural lymphatic system have not previously been described in the literature. However, with previous reports indicating a greater prevalence of ePVS and higher perfusion in males than females, we could presume that the glymphatic system may differ between males and females [23,24,25]. Accumulating evidence has suggested sex differences in relation to cognitive decline, thus supporting this notion [26,27]. Another possible explanation for these findings is the relative neuroprotective effects of estrogen and progestin [28], which may lead to preserved dural lymphatic function in female patients even after they have undergone the aging process. However, future research is required to evaluate whether men are at a greater risk of glymphatic dysfunction.

In our study, contrast filling in the peri-sinus lymphatic space after approximately 3–4 min of intravenous gadolinium administration showed rapid migration of the gadolinium from the blood vessels to the dural lymphatic vessels. The rapid enhancement of the dural lymphatics in our study can be explained as follows. Unlike the cerebral blood vessels, dural blood vessels lack a blood–meningeal barrier, and physiological dural enhancements are commonly seen on conventional gadolinium-enhanced T1WI [29]. Therefore, from the dura, small intravascular molecules like gadolinium may extravasate under a pressure gradient, drain into the lymphatic capillaries via a loose lymphatic endothelium, and finally drain into the dural lymphatic vessels and peri-sinus lymphatic space [8,30].

Recent animal and human studies have shown that intrathecal gadolinium injections lead to peri-sinus contrast enhancement in dural lymphatics visible on MRI at 50–200 min after intrathecal gadolinium injection; this finding supports the assertion that there is a link between the glymphatic system and MR-visible dural lymphatics [10,31,32]. Therefore, the direct assessment of the dural lymphatics via MRI may offer an in vivo evaluation of the glymphatic system and further study is required to explore MRI-visible dural lymphatics and their relationships with the glymphatic system.

While the present study has significant strengths, several limitations warrant further discussion. First, this study’s retrospective design included a clinically normal population with suspected brain metastases, and this may have unintentionally affected the results due to potential factors related to the disease itself and/or previous treatments. Further, we excluded patients with leptomeningeal or pachymeningeal metastasis. There may be micro- or subclinical metastases that may also affect the visualization of dural lymphatics. Accordingly, the results of this study should be interpreted with caution because of potential bias due to a disease cohort, and further studies incorporating a healthy population are required. Finally, we were unable to evaluate the association between MRI-visible dural lymphatics and neurological diseases. Additional studies including these diseased populations are needed to test the use of imaging in detecting dural lymphatic dysfunction.

In conclusion, the present study found increased peri-sinus lymphatic volume to be associated with advanced age. We also uncovered that the peri-sinus dural lymphatic space volume differs depending on sex. These findings hold potential clinical implications for the imaging of dural and glymphatic dysfunctions in numerous neurological disorders.

## Figures and Tables

**Table 1:** Patient characteristics and comparisons of demographics in study population according to age.

Variable	Total Patients *n* = 165	Non-Elderly *n* = 93	Elderly *n* = 72	*p* Value
Sex (M:F)	91:74	45:48	46: 26	0.058
Age (y)	62.1 ± 10.9	54.6 ± 7.7	71.8 ± 5.2	<0.001
Underlying disease				0.028
Lung cancer	115 (69.7%)	57 (61.3%)	58 (80.6%)	
Breast cancer	20 (12.1%)	16 (17.2%)	4 (5.6%)	
Other	20 (12.1%)	12 (12.9%)	8 (11.1%)	
None	10 (6.1%)	8 (8.6%)	2 (2.8%)	
Hypertension	48 (29.1%)	15 (16.1%)	33 (45.8%)	<0.001
Diabetes mellitus	36 (21.8%)	17 (18.3%)	19 (26.4%)	0.144
Previous chemotherapy	61 (37.0%)	31 (33.3%)	30 (41.7%)	0.271
Total Fazekas score	2.1 ± 1.4	1.5 ± 0.9	2.9 ± 1.6	<0.001
ePVS-BG (+)	18 (10.9%)	3 (3.2%)18	15 (20.8%)	<0.001
ePVS-CSO (+)	60 (36.4%)	21 (22.6%)	39 (54.9%)	<0.001
Intracranial volume (cm^3^)	1401.1 ± 131.2	1402.0 ± 140.3	1399.9 ± 119.3	0.918
Normalized dural lymphatic volume (mm^3^)	4028.0 ± 1529.8	3726.5 ± 1513.9	4417.6 ± 1470.6	0.004

ePVS-BG=enlarged perivascular space in the basal ganglia. ePVS-CSO=enlarged perivascular space in the centrum semiovale.

**Table 2:** Results of univariate and multivariate linear regression analyses to determine factors associated with peri-sinus lymphatic volume.

Variables	Univariate		Multivariate	
	β Coefficient	*p*-Value	β Coefficient	*p*-Value
Age	34.0 (12.9–55.1)	0.002	28.4 (5.7–51.2)	0.015
Male sex	1158.1 (719.0–1597.3)	<0.001	672.4 (109.9–1234.9)	0.019
DM	197.9 (−372.3 to 768.3)	0.494	-	-
HTN	469.3 (−44.9 to 983.5)	0.073	−2.8 (−517.6 to 512.0)	0.991
ePVS-CSO (+)	510.2 (26.8–993.6)	0.039	0.415 (−490.1 to 491.0)	0.999
ePVS-BG (+)	522.5 (−229.8 to 1274.8)	0.172	-	-
Intracranial volume (cm^3^)	3.9 (2.2–5.6)	<0.001	2.3 (0.16–4.4)	0.035
Brain metastasis	−86.3 (−570.4 to 397.4)	0.724	-	-
Cancer type				
None	-	-	-	-
Lung cancer	−67.4 (−1052.4 to 917.6)	0.893	-	-
Other cancer	−715.1 (−1771.4 to 341.2)	0.183	-	-
Previous Chemotherapy	−313.5 (−799.7 to 172.8)	0.205	-	-

DM = diabetes mellitus; HTN = hypertension; ePVS-CSO = enlarged perivascular space in centrum semiovale; ePVS-BG = enlarged perivascular space in basal ganglia.
